# Microstructure-Reconfigured Graphene Oxide Aerogel Metamaterials for Ultrarobust Directional Sensing at Human–Machine Interfaces

**DOI:** 10.1021/acs.nanolett.4c03706

**Published:** 2024-09-11

**Authors:** Yuhao Wang, Zhuofan Qin, Ding Wang, Dong Liu, Zibi Wang, Abdullatif Jazzar, Ping He, Zhanhu Guo, Xue Chen, Chunjiang Jia, Ximin He, Xuehua Zhang, Ben Bin Xu, Fei Chen

**Affiliations:** † School of Chemical Engineering and Technology, Xi’an Jiaotong University, No. 28, Xianning West Road, Xi’an, Shaanxi 710049, P. R. China; ‡ Mechanical and Construction Engineering, Faculty of Engineering and Environment, Northumbria University, Newcastle upon Tyne NE1 8ST, U.K.; § Department of Materials Science and Engineering, University of California, Los Angeles (UCLA), Los Angeles, California 90095, United States; ∥ Offshore Renewable Energy Catapult, Offshore House, Albert Street, Blyth NE24 1LZ, U.K.; ⊥ Department of Chemical and Materials Engineering, University of Alberta, Edmonton, Alberta T6G 1H9, Canada

**Keywords:** aerogel metamaterials, microstructure reconfiguration, buckling, pressure sensor, human−machine interface

## Abstract

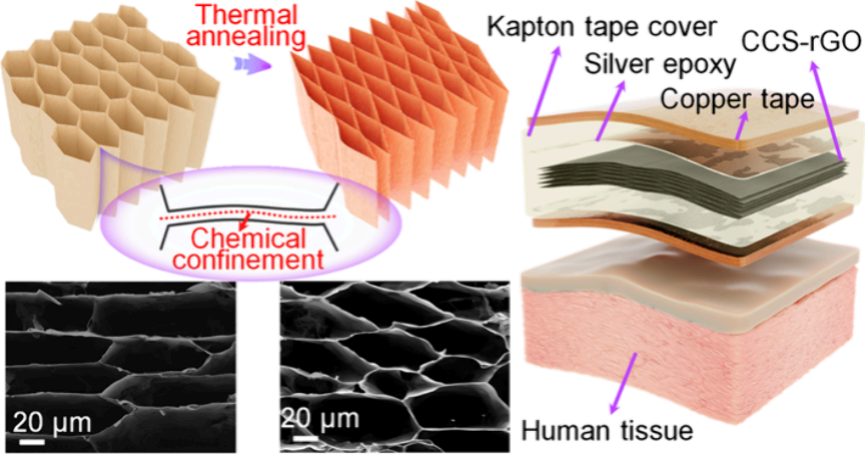

Graphene aerogels hold huge promise for the development of high-performance pressure sensors for future human–machine interfaces due to their ordered microstructure and conductive network. However, their application is hindered by the limited strain sensing range caused by the intrinsic stiffness of the porous microstructure. Herein, an anisotropic cross-linked chitosan and reduced graphene oxide (CCS-rGO) aerogel metamaterial is realized by reconfiguring the microstructure from a honeycomb to a buckling structure at the dedicated cross-section plane. The reconfigured CCS-rGO aerogel shows directional hyperelasticity with extraordinary durability (no obvious structural damage after 20 000 cycles at a directional compressive strain of ≤0.7). The CCS-rGO aerogel pressure sensor exhibits an ultrahigh sensitivity of 121.45 kPa^–1^, an unprecedented sensing range, and robust mechanical and electrical performance. The aerogel sensors are demonstrated to monitor human motions, control robotic hands, and even integrate into a flexible keyboard to play music, which opens a wide application potential in future human–machine interfaces.

Aerogels, first developed by Samuel Kistler^1^ in the 1930s, have unique features such as high porosity,^2−4^ low density,^5−7^ exceptional physical,^2^ chemical,^8^ and thermal^9^ characteristics, etc., which enable their application in thermal insulation,^9^ catalysis,^8^ biomedicine,^10^ and electronics and sensors.^3,11^ Among them, the graphene aerogel (GA) and its derivatives possess unique interconnected structures^5,12,13^ at the microscopic^6,14^ and macroscopic scales.^9,15,16^ The captivating mechanical, thermal, and electrical properties unlock its potential as the pressure sensor for robotics,^2^ human body detection,^3^ stress mapping,^11^ etc. However, the disordered and poorly connected structure of GA usually collapses under large compression,^17^ while the process for creating resilient graphene oxide (GO) is complex and energy-intensive.^18,19^ A facile and cost-effective realization of elastic GO and reduced graphene oxide (rGO) aerogels with a unique structure–property relationship is highly desired.

Metamaterials represent a class of synthetic materials with disruptive properties that cannot be found in nature.^20^ Via manipulation of the composition, structure, and dynamics, GA/graphene metamaterial is endowed with negative thermal expansion^21^ and unusual mechanical properties.^22,23^ The conventional process for directly producing a metamaterial building block, including nanoimprinting,^24^ laser writing,^25^ electron beam lithography,^26^ etc., is usually costly. A recent research focus is transforming a building block less expensively for the optimization of the auxetic response,^27−29^ modification of the photonic properties,^30^ or generation of a three-dimensional (3D) configuration from a two-dimensional (2D) template.^31^ Those attempts also involve complicated processes and excessive resources; an efficient way to reconfigure the structure for GA metamaterials is still lacking.

Herein, we propose a facile approach for producing a reduced graphene oxide (CCS-rGO) aerogel metamaterial by programming a structural reconfiguration from the honeycomb structure into an ordered buckling network on the dedicated cross-section plane. The reconfigured CCS-rGO presents a unique elasticity because the buckling network withstands large directional compression and an ultrarobustness with a strength retention of 76.2% after 20 000 cycles under a directional compressive strain of 0.7. The reconfigured CCS-rGO aerogel is designed in multimodal sensing prototypes to demonstrate its potential in human–machine interfaces.

The fabrication of the reconfigured CCS-rGO aerogel metamaterial is illustrated in Figure 1a–d. A temperature gradient was used to induce directional ice crystal growth^32^ in a chitosan/graphene oxide (CS/GO) solution. After freeze-drying,^33,34^ a honeycomb microstructure was generated in the CS-GO aerogel with various interactions [i.e., cross-linking, hydrogen bonding, and electrostatic interactions (Figure 1e,f)],^3^ where the GO aerogel without chitosan exhibits a random porous morphology (Figure 1g). The CS-GO aerogel was annealed at 180 °C for 3 h to re-reconfigure the honeycomb structure (Figure 1h) to a buckling network (Figure 1i).

**Figure 1:**
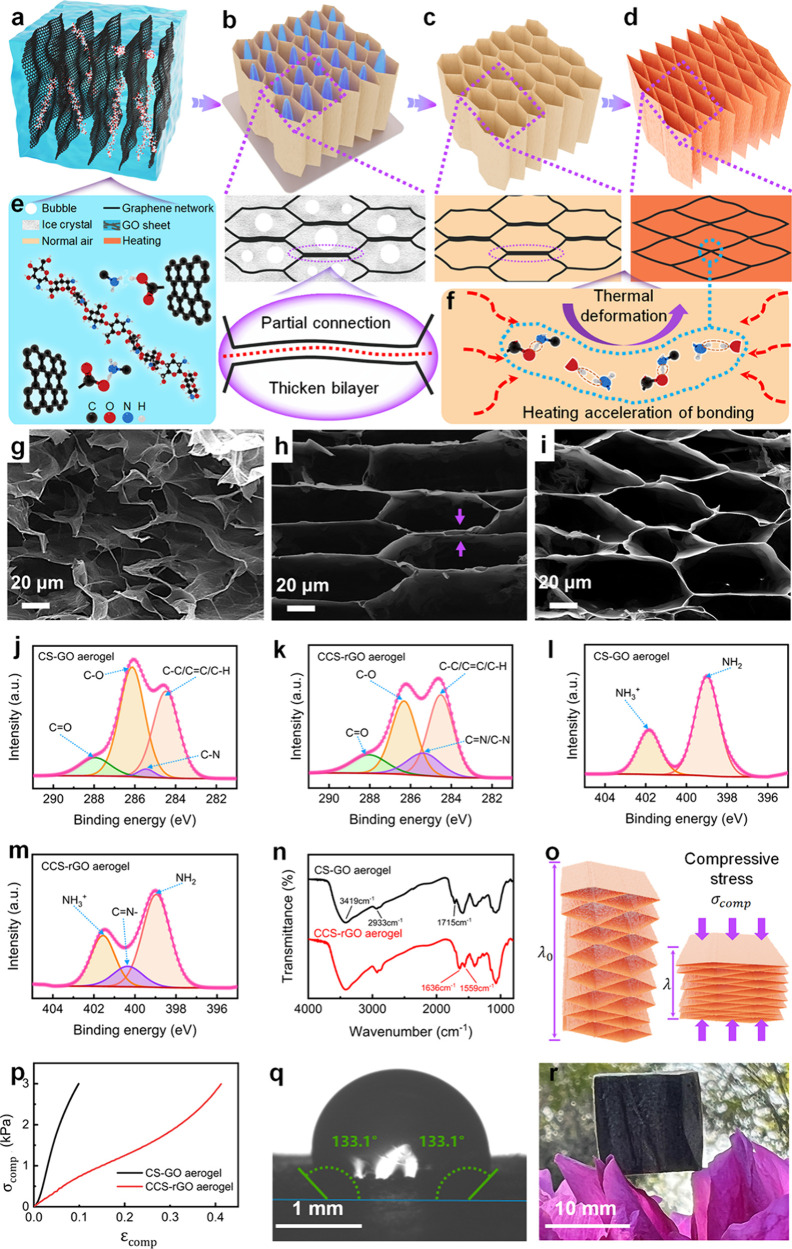
Fabrication and characterization of reconfigured CCS-rGO aerogel metamaterials. (a–d) Schematic illustration of the fabrication of CCS-rGO aerogels. (a) Mixing of GO and chitosan in water. (b) Directional freezing to generate a cross-linked GO network. (c) Freeze-drying to obtain the CS-GO aerogel. (d) Thermal annealing to achieve CCS-rGO with a reconfigured microstructure. (e) Chemical components and interactions for chitosan and GO during synthesis. (f) Chemical cross-links that form between GA and CS during annealing. Microstructure of (g) GO without chitosan, (h) CS-GO, and (i) the CCS-rGO aerogel. (j and k) C 1s spectra and (l and m) N 1s spectra of the CS-GO and CCS-rGO aerogels. (n) FTIR spectra of the CS-GO and CCS-rGO aerogels. (o) Illustration of the deformation of a CCS-rGO material under uniaxial compression. (p) Compressive stress–strain curves of CS-GO and CCS-rGO. (q) Large contact angle of water on a CCS-rGO aerogel. (r) Snapshot of the lightweight CCS-rGO aerogel on flower petals.

The results X-ray photoelectron spectroscopy (XPS) (Figure 1j,k) reveal the C 1s spectral profile of the CS-GO aerogel that can be resolved into C=O, C–O, C–N, and C–C/C–H with binding energies of 288.0, 286.1, 285.4, and 284.5 eV, respectively.^35−38^ In comparison, the C 1s profile of the CCS-rGO aerogel shows an energy shift between 285.4 and 284.5 eV, due to the formation of a C=N bond by chemical cross-linking and reduction of GO. The Raman spectra in Figure S1 also illustrate the reduction of GO with an increase in *I*_D_/*I*_G_ from 0.91 for GO to 1.54 for rGO.^39−41^ The N 1s spectral profile of the CS-GO aerogel (Figure 1l) shows the clear presence of the -NH_3_^+^ group at 401.8 eV and the -NH_2_ group at 399.0 eV.^42,43^ A characteristic C=N- group at 400.3 eV is identified for the CCS-rGO aerogel (Figure 1m). In addition, the Fourier transform infrared (FTIR) spectra (Figure 1n) of the CCS-rGO aerogel show a peak at 1636 cm^–1^ with a shoulder at 1559 cm^–1^ attributed to the formation of the C=N and C=C bonds, while the peak at 1715 cm^–1^ vanished, which resulted from the completion of chemical cross-linking.

Both XPS and FTIR results verify the cross-linking with the Schiff base reaction between the amino groups on CS and the aldehyde groups of glutaraldehyde.^44^ Thermal annealing (Figure 1f) completes the post-cross-linking reaction between CS and GA.^45^ Upon the formation of the honeycomb microstructure and prior to thermal treatment, it is postulated that the upper and lower edges of the hexagonal cell exhibit incomplete connectivity. As such, a bilayer is formed in these regions (bold line in panels b and c of Figure 1), as evidenced in Figure 1h.

The mechanical properties of aerogels are evaluated (Figure 1o,p). The compressive strain (*ε*_comp_) is defined as *ε*_comp_ = (λ_0_ – λ)/λ_0_. Under a compressive stress (*σ*_comp_) of 3 kPa, an *ε*_comp_ of 0.41 is achieved for the CCS-rGO aerogel, ∼3-fold greater than that of the CS-GO aerogel. After annealing, the cross-linked structure unlocks a hyperelastic feature. A water contact angle of 133.1° in Figure 1q indicates the hydrophobic nature of the CCS-rGO aerogel. In contrast, the contact angle on the CS-GO aerogel is ∼0° (Figure S2) and the water droplet is absorbed by the CS-GO aerogel within 0.95 s (Movie S1), due to the superhydrophilicity and porous structure. This hydrophilicity-to-hydrophobicity transition can be attributed to dehydration and removal of the surface hydroxyl group during annealing. The elemental analysis in Figure S3 shows that the level of carbon increases while the levels of oxygen and hydrogen decrease after annealing. The density of the CCS-rGO aerogel is calculated to be ∼0.0265 g/cm^3^, enabling it to stay atop the flower petals (Figure 1r).

The microstructure reconfiguration of aerogels was assessed by X-ray microtomography (MicroCT). The 3D reconstructed image (Figure 2a,e) reveals a typical honeycomb shape for the CS-GO aerogel at 25 °C, which remains intact at 140 °C (Figure 2b,f) with a lamellar layer (white arrows). An explicit buckling state is captured on the *X*–*Z* plane (yellow arrow) of the aerogel annealed at 180 °C (Figure 2c,g) with an excessive distortion on the network appearing at 220 °C (Figure 2d,h). The reconfiguration mainly occurs on the *X*–*Z* plane, as the lamellar layers along the *Y* direction (Figure 2i) have fewer cross-linked stress localizations. Dehydration and condensation are normally considered as factors for this structural transformation of the GO aerogel during annealing, decreasing the weight and volume fraction of the aerogel to form buckles.^12,46^ However, this is not the case here as our work was conducted at ∼180 °C, which is lower than the temperature range in their research (≥700 °C). Thermogravimetric analysis (TGA) of CS, GO, and CS-GO aerogels (Figure S4), indicates a limited weight loss for CS-GO (<2%), far less than the reported value (∼70%).^12^ Thus, the reconfiguration of the microstructure is driven by a thermal mechanical effect rather than dehydration.

**Figure 2:**
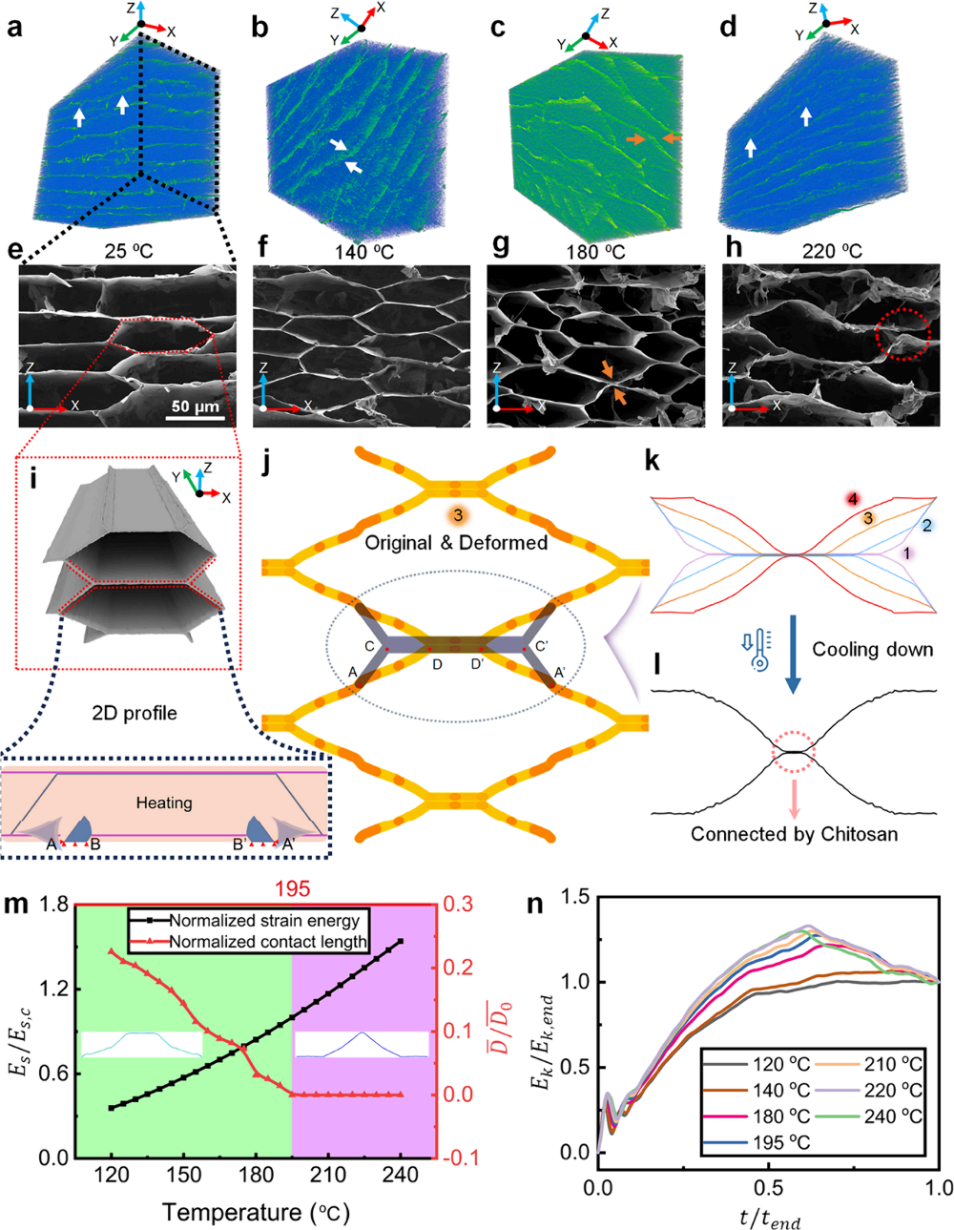
Mechanics of planar microstructure transformation in the cross section. (a–d) MicroCT images and (e–h) SEM images of microstructure for annealing temperatures of 25, 140, 180, and 220 °C, respectively. (i) 3D illustration and 2D FEA model. (j) Comparison of the initial (gray) and reconfigured (yellow) microstructure at *t*/*t*_0_ = 0.9. (k) Results of the simulation of thermal buckling by the half-cell honeycomb microstructure with different time spans. Labels 1–4 represent the state at *t*/*t*_0_ values of 0.3, 0.6, 0.9, and 1, respectively. (l) Results of the simulation of cooling after heating. (m) Phase diagram of buckled honeycomb structure (green) and buckling structure (purple) as a function of temperature. (n) Evolution of normalized kinetic energy *E*_k_/*E*_k,end_ with time ratio *t*/*t*_end_ at different temperatures.

To understand the structural transformation on the *X*–*Z* plane during the thermal process, 2D finite element analysis (FEA) was performed in ABAQUS (Figure 2i–l). With an increase in temperature from 25 to 180 °C, the structural evolution is shown in Movie S2 with the deformation on a single beam, where the initial honeycomb microstructure gradually transforms (Figure 2j,k) into the buckling state. For the simulated single cell, during thermally coupled deformation, the total length of the beam before (AC + CC′ + C′A′, i.e., gray in Figure 2j) and after heating (AD + DD′ + D′A′, i.e., orange in Figure 2j) is decreased due to the negative expansion coefficient and the localized confinement by chitosan cross-linking bonding, therefore decreasing the normalized contact length (CC′) simultaneously. As such, the honeycomb structure gradually changes into a buckling network. During cooling, the shape remains on the microstructure (Figure 2l), which is comparable to the experimental findings showing that small-scale buckles also appeared.

A phase diagram is constructed by plotting the normalized contact length *D̅*/*D*_0_ of two half-cells (*D̅* = DD′ and *D*_0_ = CC′, DD′, and CC′ are as shown in Figure 2j) in the range of 120–240 °C (Figure 2m). The increased temperatures correspond to a diminished contact length, ultimately culminating in the delamination of the two cells. In the simulation, the threshold temperature demarcating the two phases is identified as 195 °C, which aligns with a discernible surge in the normalized kinetic energy variation in *E*_k_/*E*_k,end_ in Figure 2n (*E*_k,end_ represents the end kinetic energy in the simulation). It is noteworthy that the normalized strain energy result *E*_s_/*E*_s,c_ in Figure 2m (*E*_s,c_ is the strain energy at the critical point of phase change) exhibits a linear increase in tandem with the increase in temperature, and the deformation in the *Y* direction is negligible in Figure S5, which suggests a reconfiguration primarily occurring in the *X*–*Z* plane during annealing.

Because the microstructure in the *Z*–*X* plane presents an explicit anisotropic feature (Figure 2a–h), we conduct uniaxial compression tests along the *Z* and *X* axes to verify the directional mechanical properties at different *ε*_comp_ values (Figures S6 and S7). CCS-rGO aerogels compressed along the *Z* axis exhibit a *σ*_comp_ of 20.39 kPa at an ε_comp_ of 0.7, with 98.8% stress retention after 10 cyclesn and a *σ*_comp_ of 2.78 kPa at an *ε*_comp_ of 0.3, with 95.3% retention after 10 000 cycles, showing clear anisotropy. Even after 20 000 cycles, scanning electron microscopy (SEM) images reveal well-maintained buckling structures. In comparison, the compression in the *X* axis results in a higher *σ*_comp_ (42.33 kPa) and lower stress retention (73.3%) after 10 cycles (at an *ε*_comp_ of 0.7) and a higher *σ*_comp_ of 11.04 kPa and a lower retention of 61.95% after 10 000 cycles (at an *ε*_comp_ of 0.3) with damaged structure observed. Comparatively, RCS-rGO aerogels (from random freezing) show a significant stress loss of 81.09% after 10 000 cycles, due to the severe structural failures. Furthermore, we compare the fatigue resistance (Figure S8) at increasing temperatures (200, 220, and 240 °C). All aerogels present good stress retention after 20 000 cycles. Stress retention values of aerogels prepared at 200, 220, and 240 °C are 85.1%, 86.9%, and 87.3%, respectively. However, it is found that the *σ*_comp_ of an aerogel prepared at 240 °C did not change over an *ε*_comp_ range of 0–0.05 at the 20 000th cycle (Figure S8c) due to the excessive structural distortion (Figure 2d,h).

Further studies of the elasticity and fatigue resistance of the CCS-rGO aerogel are undertaken along the *Z* axis. Remarkably, no explicit damage is found (Figure 3a,b) even after 20 000 compression cycles. The stress–strain curves of the CCS-rGO aerogel in Figure 3c (*ε*_comp_ of ≤0.5) and Figure S9 (*ε*_comp_ of ≤0.9) display a sharp rise with 50% compression. *σ*_comp_ increases uniformly with *ε*_comp_ without a stress plateau, indicating its structural stability. The CCS-rGO aerogel can undergo a maximum *ε*_comp_ of 0.95 and a maximum *σ*_comp_ of 146.36 kPa. We next assessed the microstructure of the CCS-rGO aerogel at *ε*_comp_ values of 0%, 30%, and 50% with SEM (Figure S10). The vertical length of a buckling cell decreased by 5.13% from 0% to 30% strain and by 5.43% from 30% to 250% strain. Notably, the macroscopic compression strain of the aerogel (from 0% to 50%) results from small strains (∼5%) in the buckling cells, where the cells can fully recover their original shape after compression, endowing the aerogel with excellent elasticity.

**Figure 3:**
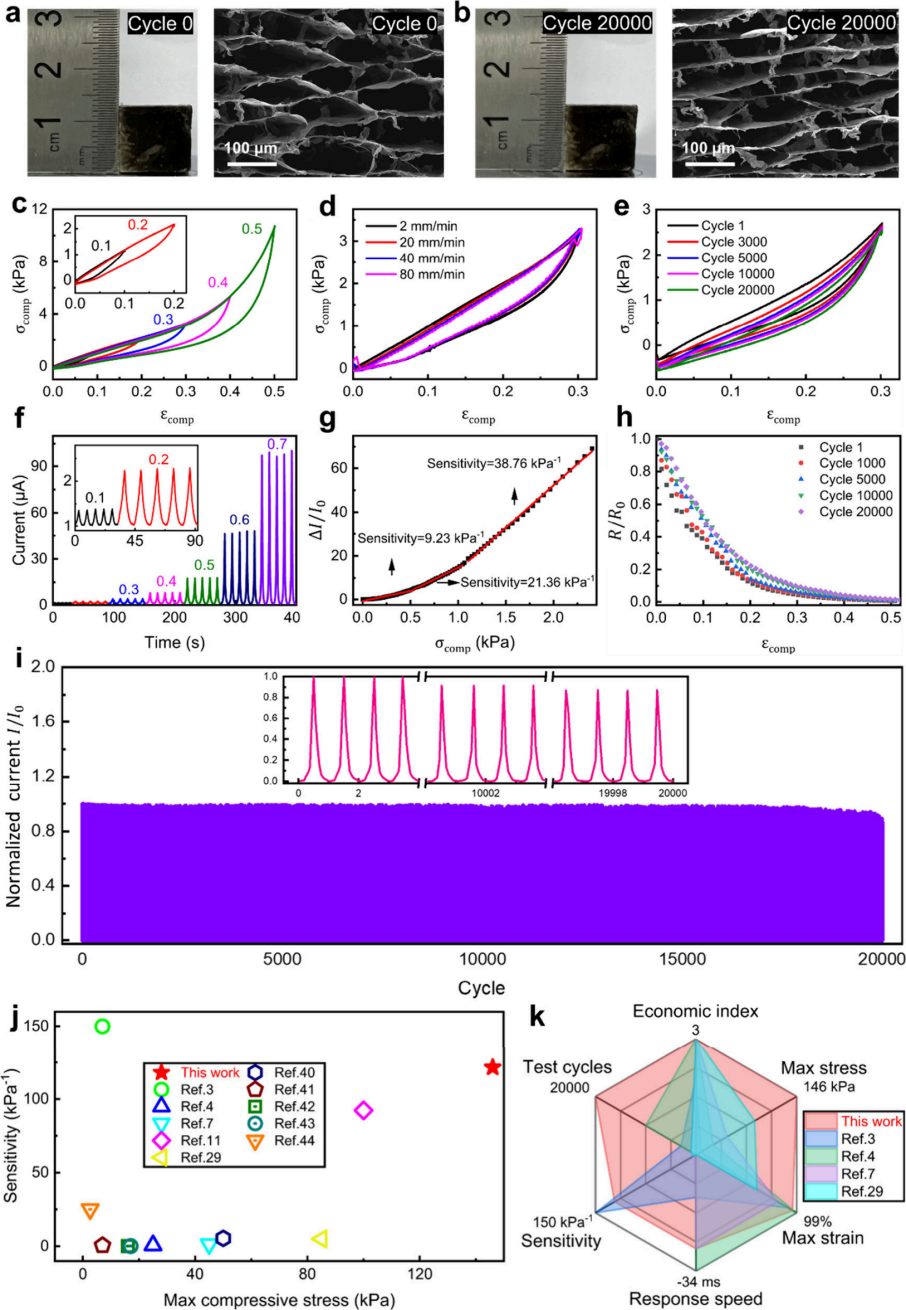
Robust directional mechanosensing performance of the CCS-rGO aerogel. Morphologies (a) before and (b) after 20 000 compressive cycles in the *Z* direction. (c) Stress–strain curves of the aerogel compressed over an *ε*_comp_ range of 0.1–0.5. (d) Stress–strain curves of the aerogel at different loading rates. (e) Stress–strain curves of the aerogel under cyclic compression with a fixed *ε*_comp_ of 0.3. (f) Current response at different levels of *ε*_comp_. (g) Sensitivity over the *σ*_comp_ range of 0–2.5 kPa. (h) Resistance variation related to *ε*_comp_ with cyclic compression. (i) Current stability for 20 000 cycles at an *ε*_comp_ of 0.5. (j) Comparison of the sensitivity and maximum compressive stress of our pressure sensor with those of existing sensors. (k) Comparison of multiple parameters, including economic index, test cycles, maximum compressive stress, maximum compressive strain, sensitivity, and response speed (to ensure the consistency of beneficial growth trends for each parameter, the response speed has been treated as a negative number), of our pressure sensor and those of other reduced graphene oxide aerogel sensors.

Figure 3d shows the stress–strain curves of the CCS-rGO aerogel at different loading rates. The CCS-rGO aerogel presents excellent stress retention of 86.2% after 20 000 cycles (Figure 3e). The energy dissipation coefficient of the CCS-rGO aerogel after 20 000 cycles is 0.225 (calculated from Figure 3e and Figure S11), which is desirable for an aerogel. It can also withstand the dynamic impact load from the impact of a stainless-steel ball (weight of ∼4 g) at an incident velocity of ∼1.4 m/s, with no visible structural damage (Movie S3). A 2D FEA is conducted to understand the mechanical behavior of aerogels under compression (Figure S12), and the result of the simulation agrees well with the experimental result (Figure 1o). Moreover, we explore the tensile properties of CCS-rGO aerogels along the *Z* axis (Figure S13) to complement the mechanical study. High durability is uncovered at a variable loading rate between 5 and 20 mm/min with 96.9% tensile stress retention after 100 tension cycles at a tensile strain of 0.15, with good retention of buckling microstructures.

The real-time current responses along the *Z* axis at different *ε*_comp_ values are plotted in Figure 3f for the CCS-rGO aerogel. A sharp and stable sensing performance is observed with a symmetric curve shape over the *ε*_comp_ range of 0.1–0.7. The sensitivity (*S*) can be calculated from the following equation:
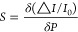
1where *I*_0_ is the initial output current, *ΔI* is the change in current due to the applied pressure, and *δP* is the change in the applied pressure.^47,48^ Under the same pressure change *δP*, a larger relative current change δ(Δ*I*/*I*_0_) corresponds to a higher sensitivity.^48^Figure 3g shows output current change Δ*I*/*I*_0_ under pressures of 0–2.5 kPa, which is related to human motion detection, and the sensitivities at different *σ*_comp_ values can be determined to be 9.23 kPa^–1^ for 0–0.5 kPa, 21.36 kPa^–1^ for 0.5–1 kPa, and 38.76 kPa^–1^ for 1–2.5 kPa. This sensitivity can be maintained for a wider sensing scale of up to 146.7 kPa pressure at an *ε*_comp_ of 95% (Figures S14 and S15), with a short response time of 60 ms.

Under a cyclic compression at an *ε*_comp_ of 0.5, the resistance response (Figure 3h) remains stable after 20 000 cycles, with a <10% loss of *R*/*R*_0_. The normalized current value (*I*/*I*_0_) remains identical for the initial 1000 cycles (Figure 3i), slightly decays, and stabilizes for the next 19 000 cycles. This robust performance can be attributed to the structural stability and elasticity enabled by the reconfigured buckling microstructure.^49^ In Figure 3j, we compare the CCS-rGO aerogel base sensor with other pressure sensors,^3,4,7,11,38,50−54^ where the CCS-rGO aerogel base sensor show an impressive overall performance with a maximum *σ*_comp_ and sensitivity. The comprehensive properties of our CCS-rGO aerogel were compared with those of other rGO aerogel pressure sensors (Figure 3k), e.g., honeycomb PI/rGO aerogel,^7^ rGO@carbon nanotubes/chitosan aerogel,^38^ etc. We also assigned an economic index to represent the cost of the materials per sensor body as follows: >$1 for index 1, $0.1–1 for index 2, and <$0.1 for index 3. Interestingly, our CCS-rGO aerogel stands out with a comprehensive performance including a relatively facile preparation method, high sensitivity, a medium response time, and wide sensing stress and strain ranges.

A CCS-rGO aerogel-based sensor was assembled in a sandwich design for bending angle detection (Figure 4a), to monitor human motions. Four different bending angles can be easily distinguished by a stepwise current curve. The mechanics of this sensor design is analyzed by following the resistance–strain curves in Figure 3h. When compressed, the buckled layer deforms, and the contact areas between neighboring layers increase, which decreases the electrical resistance. Therefore, relative resistance *R*/*R*_0_ can be expressed as a function of *ε*_comp_:
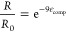
2

**Figure 4:**
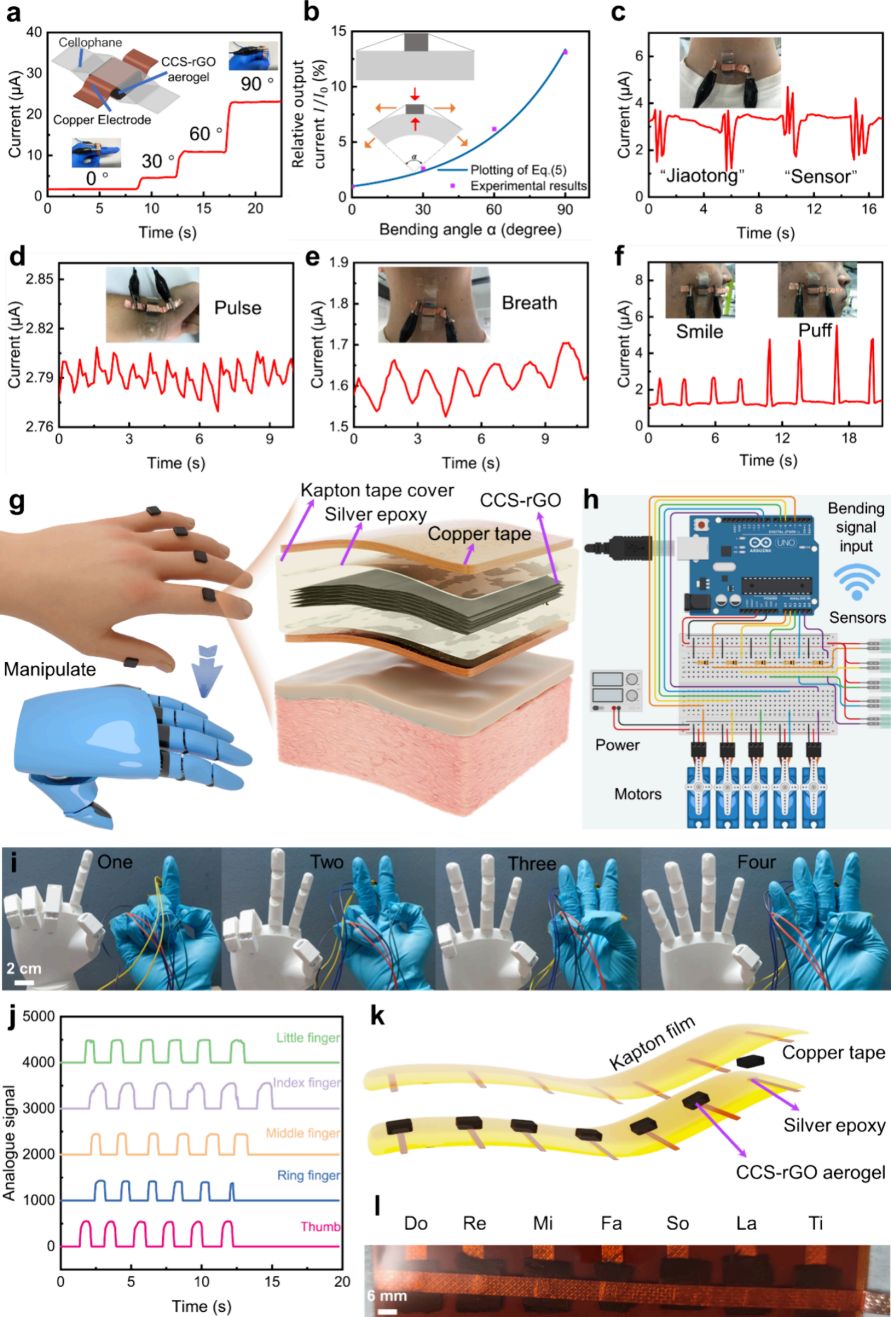
CCS-rGO aerogel-based multimodal pressure sensor for different scenarios. (a) CCS-rGO simple test cell (inserts) and angle–current response of finger bending. (b) Equivalent 2D model for the bending mechanism. Current signals of human body detection, including (c) facial expression, (d) pulse, (e) speaking “jiaotong” and “sensor”, and (f) breathing. (g) Configuration of the finger sensor for manipulating the robotic hand. (h) Schematic diagram of the human–machine remote control system. (i) Five fingers under different strains for corresponding finger synchronized gestures of a robotic hand. (j) Analogue signal values of each finger related to four gestures. (k and l) Prototype of an electrical piano based on a CCS-rGO aerogel customized array and the product.

The output current is inversely proportional to the resistance. Thus, from eq 2, we can also obtain the expression of relative current *I*/*I*_0_ as a function of *ε*_comp_:
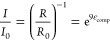
3

For the sensing cell in Figure 4a, the model of the mechanics can be simplified as a two-body structure; the aerogel is adhered to a beam that can bend (inset of Figure 4b). When the beam bends, its top surface moves upward, which imparts compressive stress on the CCS-rGO aerogel along the same direction of motion. The resulting *ε*_comp_ of the aerogel block increases with bending angle α of the deformed beam. Thus, *ε*_comp_ can be expressed as a linear increasing function of α:
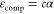
4where linear coefficient *c* depends on the geometry and mechanical properties of the sensor part. By introducing eq 4 into eq 3, we obtain the relation between relative current *I*/*I*_0_ (used as a detecting signal) and bending angle α as
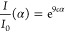
5

With a *c* of 0.0032 deg^–1^, eq 5 is plotted in Figure 4b, and good agreement with experimental data is shown. If attached to the throat, the sensor can detect the differences in the vibration of vocal cords when speaking different words, such as “jiaotong” and “sensor” (Figure 4c). The sensor can also capture other different biosignals, such as pulse^55,56^ (Figure 4d), breathing (Figure 4e), and changes in facial expression (e.g., smile and puff in Figure 4f).

We next conduct a failure test in the *X* direction for the sensor (Figure S16) by measuring the analogy signal over time. Sensitivity in the *X* pressing direction is reduced by one-third compared to that in the *Z* direction, and the signal curve is not smooth during the first 10 cycles. After 100 cycles in the *X* direction, the curve becomes smooth with a sensitivity at one-third of the normal level.

Leveraging the extraordinary mechanical properties and exceptional mechanosensing capabilities, we assembled a sensor by encapsulating the aerogel between two layers of silver epoxy/copper tape with a Kapton tape cover (Figure 4g). We then utilize the sensor as a human–machine interface to control a robotic hand. To showcase the versatility of this technology, an Arduino board, voltage dividers, and a 3D-printed custom robotic hand, actuated by servo motors, are assembled (Figure 4h). The system allows the precise control of the gestures of a robotic hand (Figure 4i). The dynamic sensing data are recorded in real time (Movie S4) to demonstrate the accurate sensing of the external pressure. Individual sensing curves for each finger are summarized in Figure 4j, specifying the distinct and discernible signals produced by our CCS-rGO aerogel sensors.

To further demonstrate the designability of the sensor array with CCS-rGO aerogels, we have assembled a flexible electrical piano, composed of seven CCS-rGO aerogels configured with silver epoxy, copper tape, and Kapton film to represent seven basic music notes in panels k and l of Figure 4, playing a short song through a buzzer in Movie S5. Also, a tactile sensing keyboard consisting of 28 CCS-rGO aerogels with copper electrodes is fabricated (Figure S17).^57^ Customized keyboard buttons corresponding to the 26 letters and two forms of punctuation are incorporated. As a demonstration, pressing the “X”, “J”, “T”, “U”, and “!” buttons in sequence reviews the curves of resistance change with time. This demonstration reveals an integrated vision for future applications based on CCS-rGO aerogels.

In conclusion, a new CCS-rGO aerogel metamaterial technology is developed by reconfiguring the microstructure of aerogel materials from a honeycomb structure to a buckling structure. The aerogel metamaterial can sustain large external compressive strains and exhibit smooth pressure–strain curves for a wider sensing range. Remarkably, the CCS-rGO aerogel can undergo 20 000 cycles of 70% compression with 86.2% stress retention, exhibit superior mechanical properties in the *Z* axis (perpendicular to the lamellar layers) direction, and have an ultrasensitivity of 121.45 kPa^–1^. A rich set of CCS-rGO aerogel metamaterial-based sensing prototypes are assembled to demonstrate their potential for human motion detection, the manipulation of a robotic hand, and the manipulation of an electrical keyboard.

## Supporting Information Available

Supplementary Experimental Section, Figures S1–S17, and Table S1 (PDF)
Deposition of a water droplet on the surface of the CS-GO aerogel (Movie S1) (MP4)
FE simulation of the thermomechanical deformation on a single beam (Movie S2) (MP4)
Dynamic impact of free-falling steel ball on the CCS-rGO aerogel (Movie S3) (MP4)
Demonstration of sensing accuracy and application as robotic hand control (Movie S4) (MP4)
Playing ‘Twinkle, Twinkle, Little Star’ with a CCS-rGO sensor-based digital keyboard (Movie S5) (MP4)
