# Supplementary material for: Microstructure-Reconfigured Graphene Oxide Aerogel Metamaterials for Ultrarobust Directional Sensing at Human–Machine Interfaces
Source: Nano Lett. 2024 Sep 11;24(38):12000–9. doi: 10.1021/acs.nanolett.4c03706 (PMC11440644; doi:10.1021/acs.nanolett.4c03706)
Supplement: Supplementary file 1 — nl4c03706_si_001.pdf [file nl4c03706_si_001.pdf]

SUPPORTING INFORMATION:

Microstructure Reconfigured Graphene Oxide Aerogel Metamaterials for Ultra-robust Directional Sensing at Human-machine Interfaces

Yuhao Wang‡[1], Zhuofan Qin‡[2], Ding Wang[2, 4], Dong Liu[1], Zibi Wang[1], Abdullatif Jazzar[3], Ping He[3], Zhanhu Guo[2], Xue Chen[2], Chunjiang Jia[4], Ximin He[3], Xuehua Zhang*[5], Ben Bin Xu*[2] and Fei Chen*[1]*

[1]School of Chemical Engineering and Technology, Xi'an Jiaotong University, No. 28, Xianning West Road, Xi'an, Shaanxi, 710049, PR China

[2]Mechanical and Construction Engineering, Faculty of Engineering and Environment, Northumbria University, Newcastle upon Tyne, NE1 8ST, UK

[3]Department of Materials Science and Engineering, University of California, Los Angeles (UCLA), Los Angeles, CA 90095, USA

[4]Offshore Renewable Energy Catapult, Offshore House, Albert Street, Blyth, NE24 1LZ, UK

[5]Department of Chemical and Materials Engineering, University of Alberta, Edmonton, Alberta, T6G 1H9, Canada

Contents

Supplementary Experimental Section....................2
Supporting Figures....................7
Supporting Table....................24
Supporting Movies....................25

Supplementary Experimental Section

Materials: Chitosan (deacetylation ≥85%, viscosity = 200 cps), L-Ascorbic acid were purchased from Shanghai Macklin Biochemical Co., Ltd. Glutaric dialdehyde (50% in water) was purchased from Shanghai Titan Scientific Co., Ltd. Glacial acetic acid was purchased from Sinopharm Chemical Reagent Co., Ltd. Graphite power was purchased from Qingdao Huatai Co., Ltd.

Fabrication of CCS-rGO aerogels and other aerogels: Graphene oxide was synthesized by improved Hummer method.[1] Mix 30 mL 6 mg/mL GO aqueous solution and 30 mL 20 mg/mL CS solution with ultrasonic and stirring for 0.5 hour to prepare a homogenous CS/GO solution. Then injected 2 mL 270 mg/mL L-ascorbic acid solution into the CS/GO solution. Afterwards, 1.6 mL of 12.5 wt.% glutaraldehyde solution was added dropwise to the mixture above, followed by transferring into a casting mould for freeze-casting and then freeze-dried to prepare CS-GO aerogel (**Figure 1**a). After heating CS-GO aerogel in vacuum oven at 180℃ for 3 hours, the CCS-rGO aerogel was obtained. For comparison purposes, the aerogel was named RCS-rGO if the mixture was prepared by random freezing instead of freeze-casting. We also prepared 8 different groups of CCS aerogels without GO, named CCS-1, CCS-2, CCS-3, CCS-4, CCS-5, CCS-6, CCS-7, CCS-8 aerogel, respectively (Table S1). 30 mL 10 mg/mL CS solution was mixed well with 0.8 mL of 12.5 wt.% glutaraldehyde solution, and then followed by freeze-casting and freeze drying to obtain

CS aerogel. In addition, we prepared GO aerogels by freeze-casting methods, and reduced GO (rGO) aerogels by L-ascorbic acid and 180℃ heating for 3 hours.

Morphological and chemical characterization: The morphologies of samples were observed by a scanning electron microscope (SEM, TESCAN, MAIA3 LMH) and a transmission electron microscope (TEM, JEM-2100Plus, JEOL Ltd., Japan). Elemental analysis was measured by Elementar (vario EL cube, Germany). The chemical structure of the materials was measured using a Fourier transform infrared (FTIR) spectroscope by attenuated total refraction mode (Nicolet iS50, Thermo Fisher Corp., America). The chemical compositions on the surface of CS-GO/CCS-rGO aerogels were studied by X-ray photoelectron spectrometer (XPS, Thermo Fisher ESCALAB Xi+, USA). Water contact angles of CS-GO/CCS-rGO aerogels were measured by contact angle goniometer (DSA100, KRUSS, Germany). Raman spectra of GO/rGO aerogel were recorded by Raman spectrometer (532 nm laser, Thermo Fisher Corp., America). The heating process was investigated by TGA (Thermogravimetric Analysis, TGA2, METTLER TOLEDO) with a heating rate of 10 ℃/min from 30℃ to 700℃ in N_2 gas.

The X-ray micro-CT (ZEISS, Xradia 610 Versa) system was used to visualize the 3D aerogel structure. X-ray micrograph images were attained with a rotation for a 360° range, using a transmission grating with a voltage of 80 kV and a power of 10 W. The total acquisition time was 3 h with the optical magnification of 20.110701 and exposure time of 1 s. The beam hardening

correction and 3D reconstruction of the x-ray micrograph were performed using the Dragonfly software.

Mechanical testing: Mechanical properties (including compression-decompression, and cyclic stability) were measured on a multifunctional mechanical tester (SUST, China) with a 30 N load cell. Rebounding ability was detected by high-speed camera (MemrecamHX-6E, NAC, Japan).

Piezoresistive properties and sensing Testing: The piezoresistive sensor was fabricated by placing CCS-rGO aerogel (12 mm x 12 mm x 8mm) between two copper conductive films (12 mm x 40 mm x 1 mm) connected by cellophane. The resistance and current were measured using a 2450 digital source meter (KEITHLEY, Tektronix Company, America).

Robotic hand and controlling: The main body and fingers of the robotic hand were produced using a 3D printer (Ultimaker S5) with general PLA filament (General PLA, Ultimaker). The four fingers were assembled using elastic string bungee cords (4mm diameter, Fantasyon, Amazon). Each finger was actuated by a fishing line (0.5mm diameter, Aidiao, Amazon) and a servo motor (Seeed Studio Grove-Servo, RS Components). The aerogel sensors were composed of CCS-rGO aerogel, silver epoxy (MG Chemicals 8331D Silver Conductive Epoxy Adhesive, RS Components), copper tapes (3M 1181 Conductive Copper Tape, 12.7mm, RS Components), enclosed by Kapton tape

(19mm, RS Components), and bound to the fingers by a tailored sleeve made from standard laboratory nitrile gloves. The controlling and signal monitoring system comprised an Arduino Uno R3 board, a breadboard, and a 10kΩ resistor as a voltage divider.

Flexible music keyboard and controlling: The flexible music keyboard was fabricated using 7 CCS-rGO aerogels (approximately 10mm x 10mm x 4mm), the same silver epoxy as mentioned earlier and custom-tailored 10mm copper tapes (as previously described). These components were sandwiched by two Kapton films (300HN, cut into a rectangular keyboard shape) and the films were affixed using double-sided tapes (5mm width, Amazon).

Finite element modelling: The commercial FE software ABAQUS is used. To simulate morphology, change by heating, the thermal-coupled deformation module is used. Since the framework of microstructures is mainly composed of GO/rGO, the thermal-mechanical properties of GO (**Table. 1**) were applied in the simulation.[2-10]

Table 1. Thermal-mechanical properties of GO for simulation.

Symbol	Meaning	Value measured in the literature	Value taken in simulation
ρ	Density[2]	(1.5~1.9) ×10^{-3} [kton/m^3]	1.5×10^{-3} [kton/m^3]
E	Elastic modulus[3]	(0.8~1.8) ×10^3 [Mpa]	1.8×10^3 [MPa]
ν	Poisson's ratio[4, 5]	-0.6 ~ +0.22	-0.6
k	Thermal conductivity[6, 7]	(5~10) [W/m·K]	7 [W/m·K]

α	Expansion coefficient[8]	$-1208\sim-67\times10^{-6}/^{0}C$	-67×10^{-4} [/K]
C_p	Specific heat capacity[9, 10]	710~1000 [J/kg·K]	710 [J/kg·K]

Supporting Figures

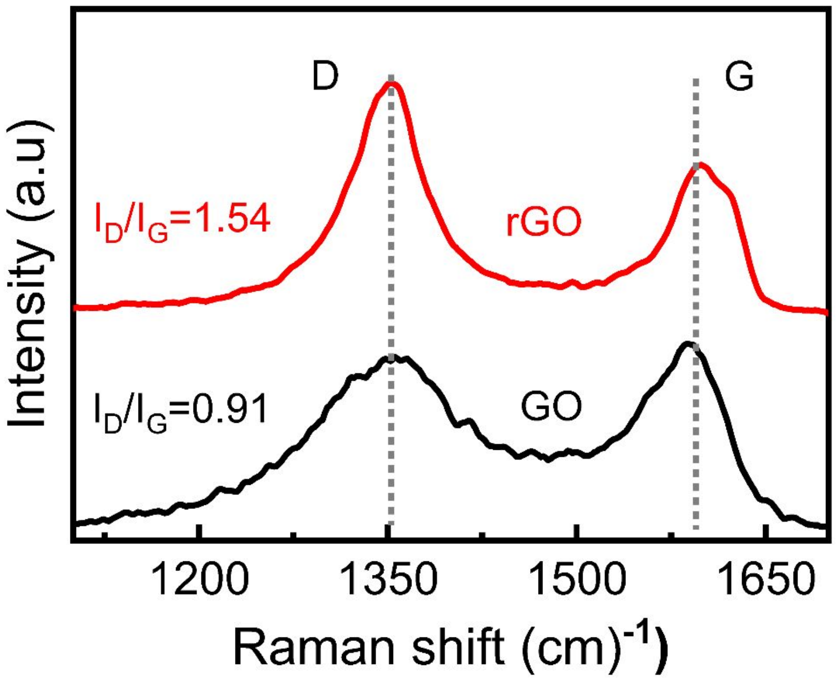

Figure S1. Raman spectroscopy of GO and rGO.

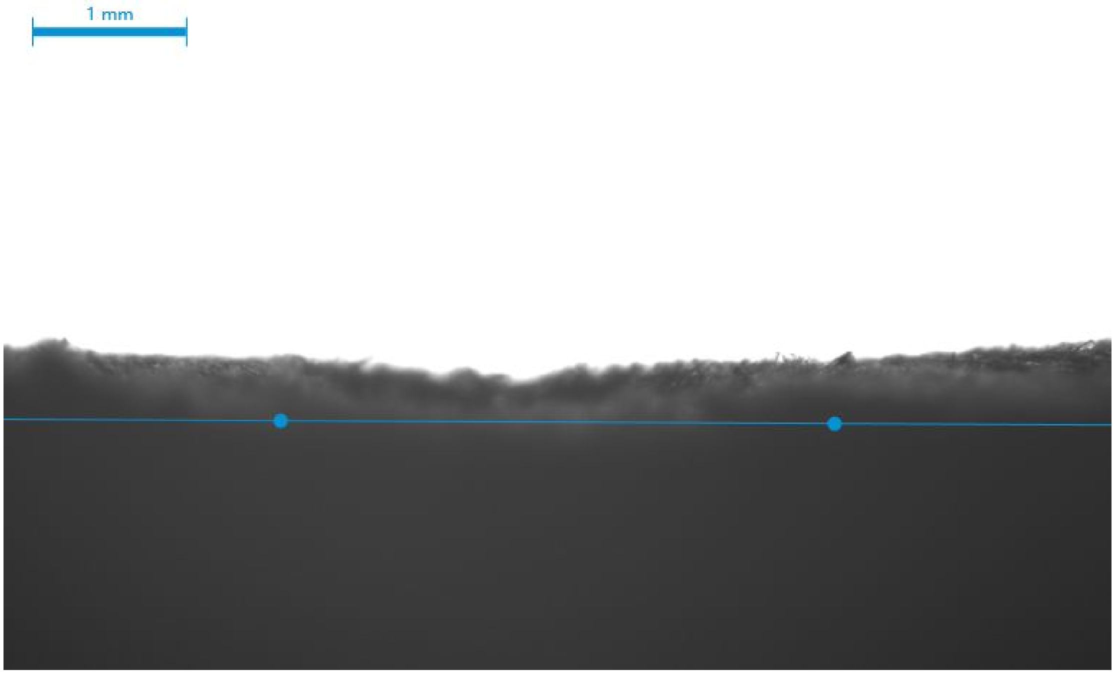

Figure S2. Water contact angle of CS-GO aerogel. The water droplet has been absorbed due to the hydrophilicity.

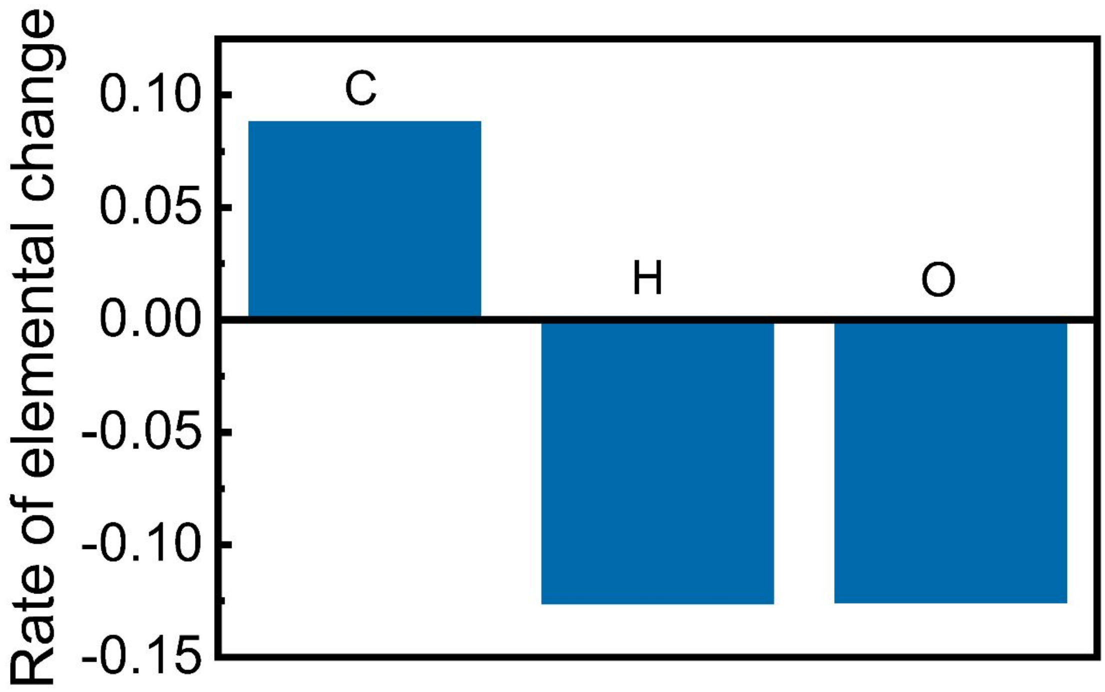

Figure S3. Elemental analysis is employed to detect the changes of elements from CS-GO to CCS-rGO and the results show that C element increases.

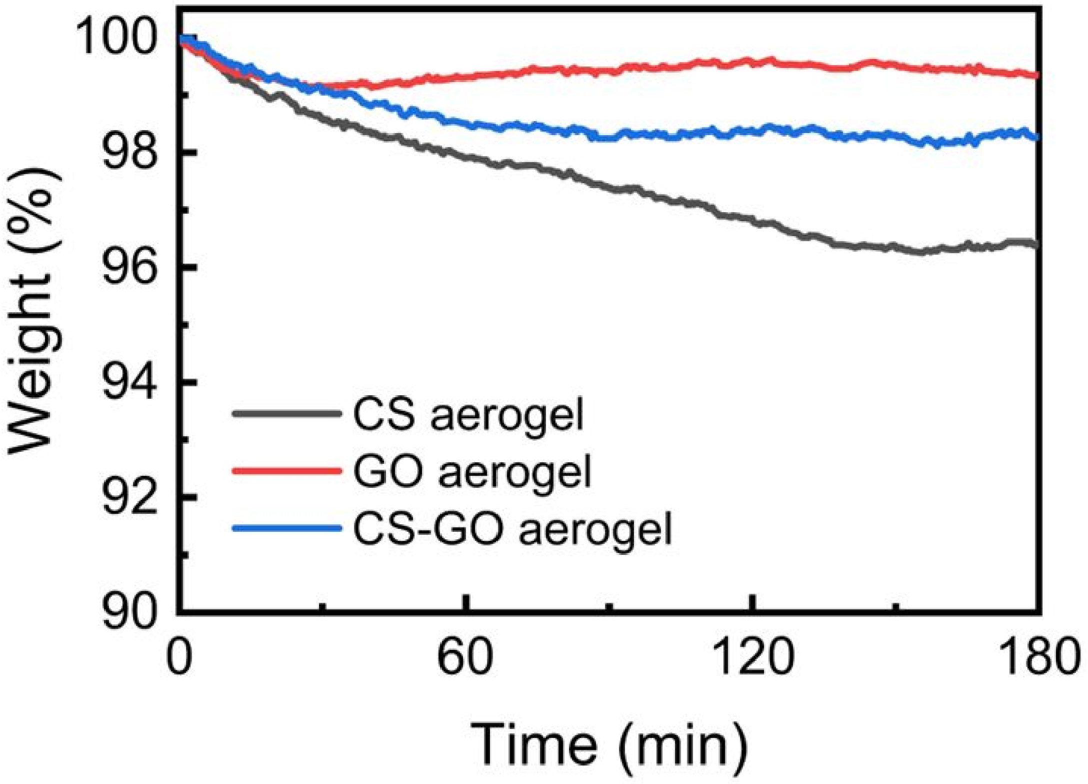

Figure S4. Thermogravimetric Analysis (TGA) for CS, GO, CS-GO aerogel at 180 ° C for 3 hours.

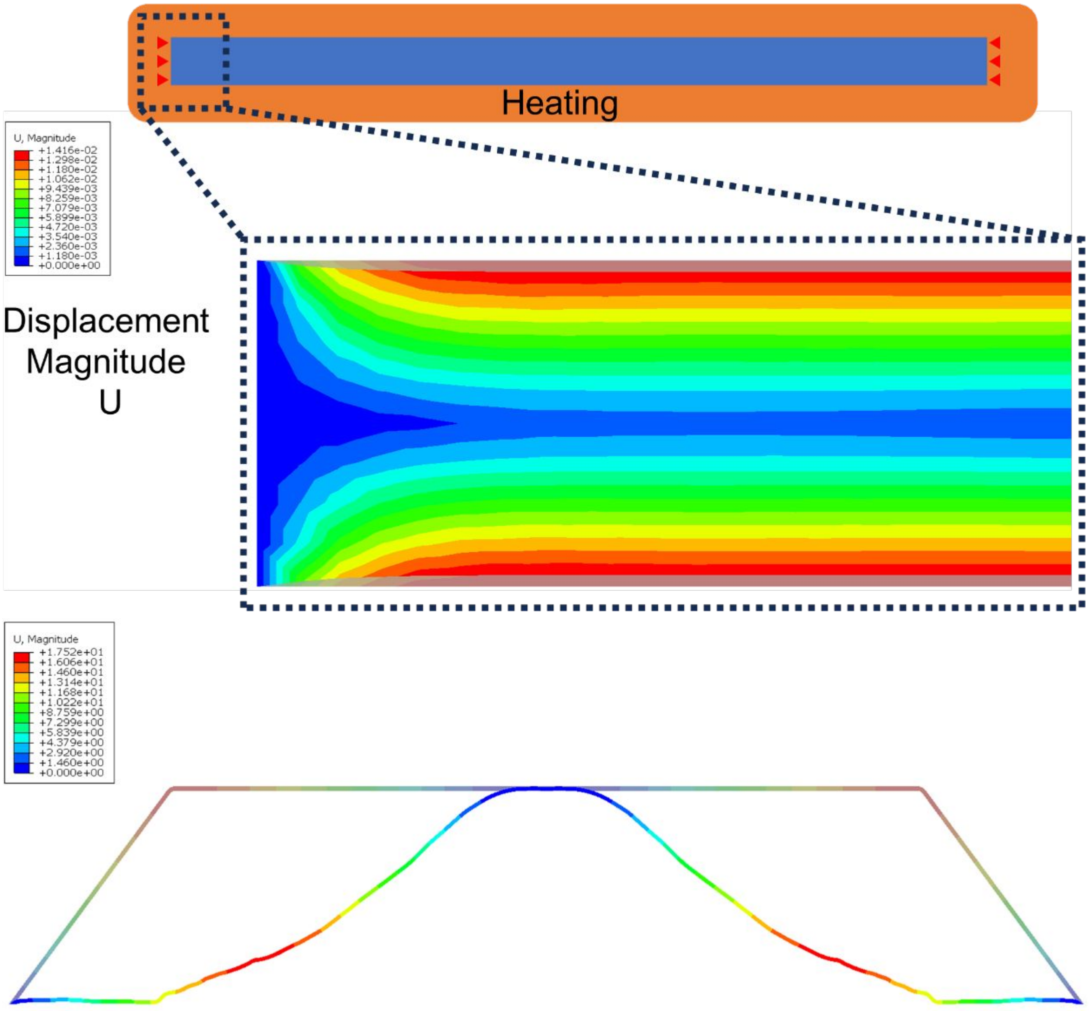

Figure S5. Comparison of displacement contour by FE simulation in Y direction of the microstructure. Top one is the profile at Y direction and corresponding magnified view of left side. The outer faded one is the original shape and no obvious deformation by the legend of displacement. Bottom one is the same deformation contour in X-Z plane. Configuration and load setting in two simulations are same and the dimensions are matched to SEM results.

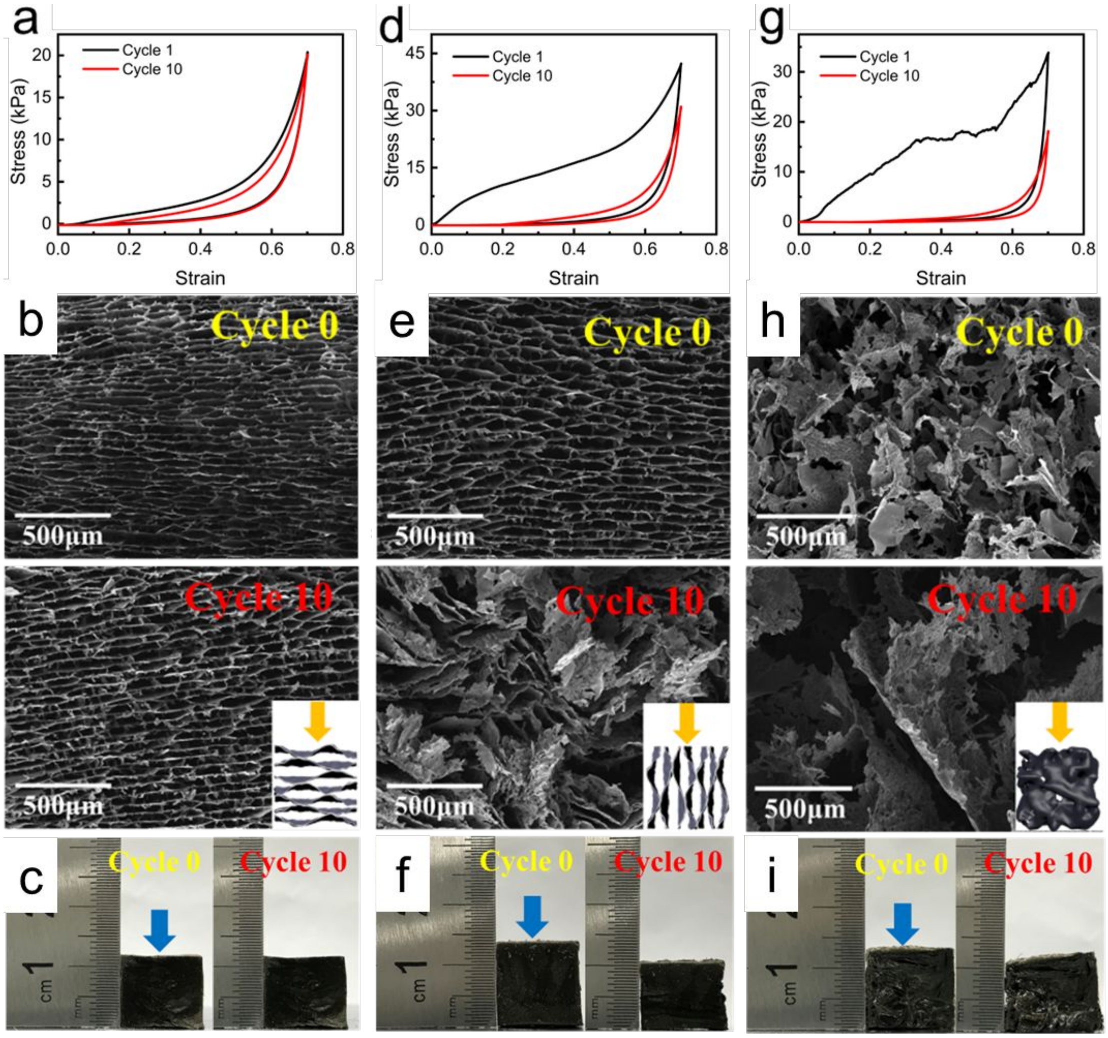

Figure S6. Anisotropic properties of the CCS-rGO by uniaxial compression. a-c) CCS-rGO in radial direction (i.e. Z direction). d-f) CCS-rGO in axial direction (i.e. X direction). g-i) RCS-rGO by compression. a,d,g) Stress−strain curves, b,e,h) SEM images and c,f,i) photographs before and after compression up to 70% strain.

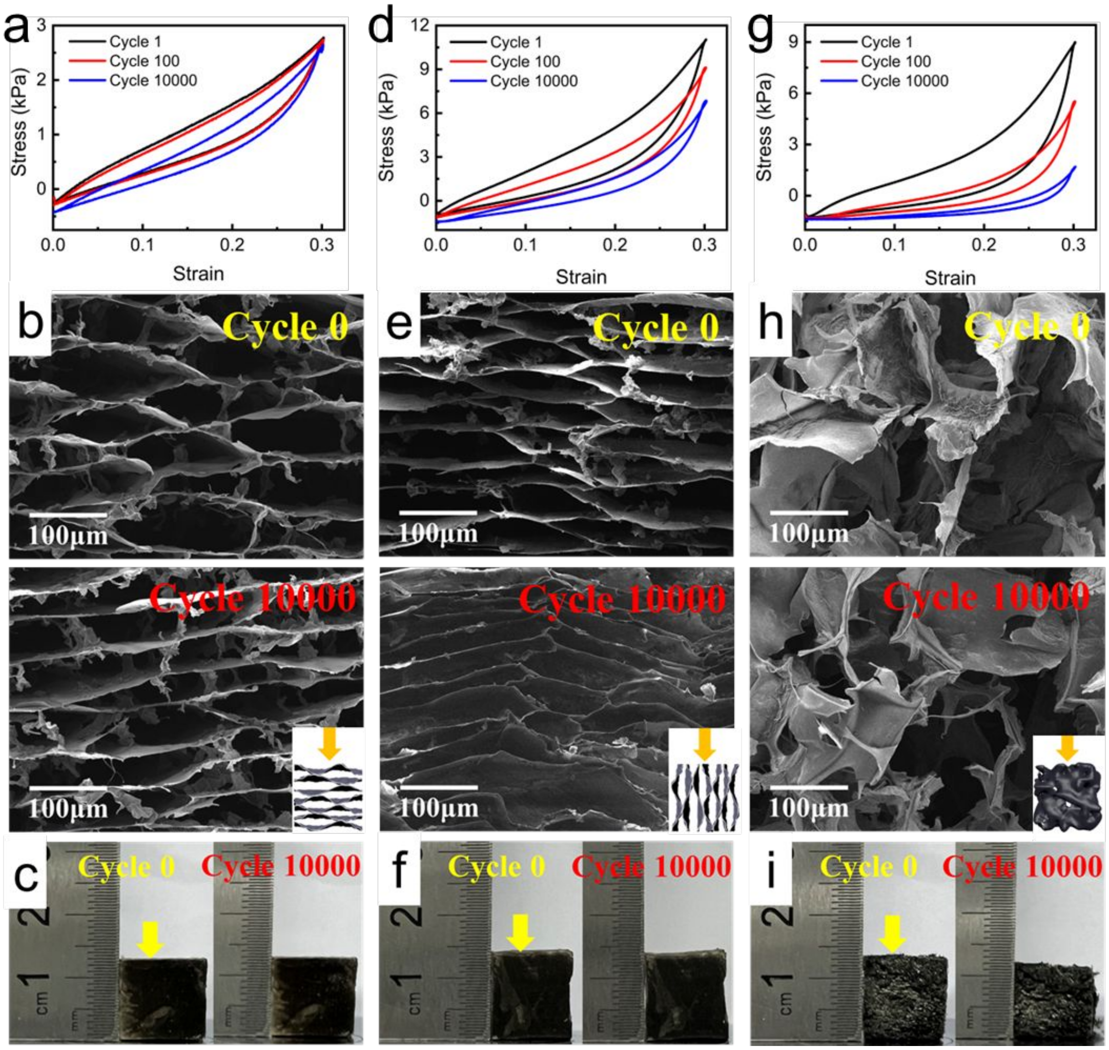

Figure S7. Anisotropic properties of the CCS-rGO by uniaxial compression. a-c) CCS-rGO in radial direction (i.e. Z direction). d-f) CCS-rGO in axial direction (i.e. X direction). g-i) RCS-rGO by compression. a,d,g) Stress−strain curves, b,e,h) SEM images and c,f,i) photographs before and after compression up to 30% strain.

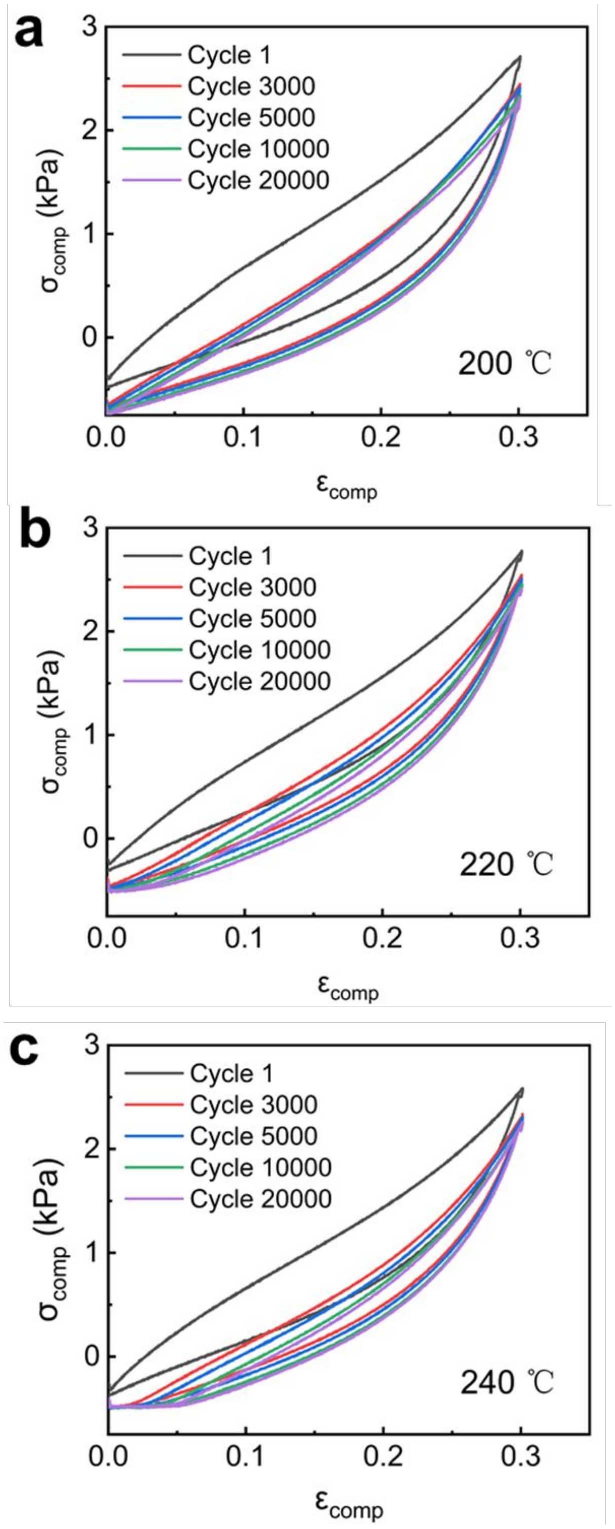

Figure S8. Stress-strain curves of different aerogels prepared at a) 200℃, b) 220℃, c) 240℃ under cyclic compression with a fixed ε_{comp} of 0.3.

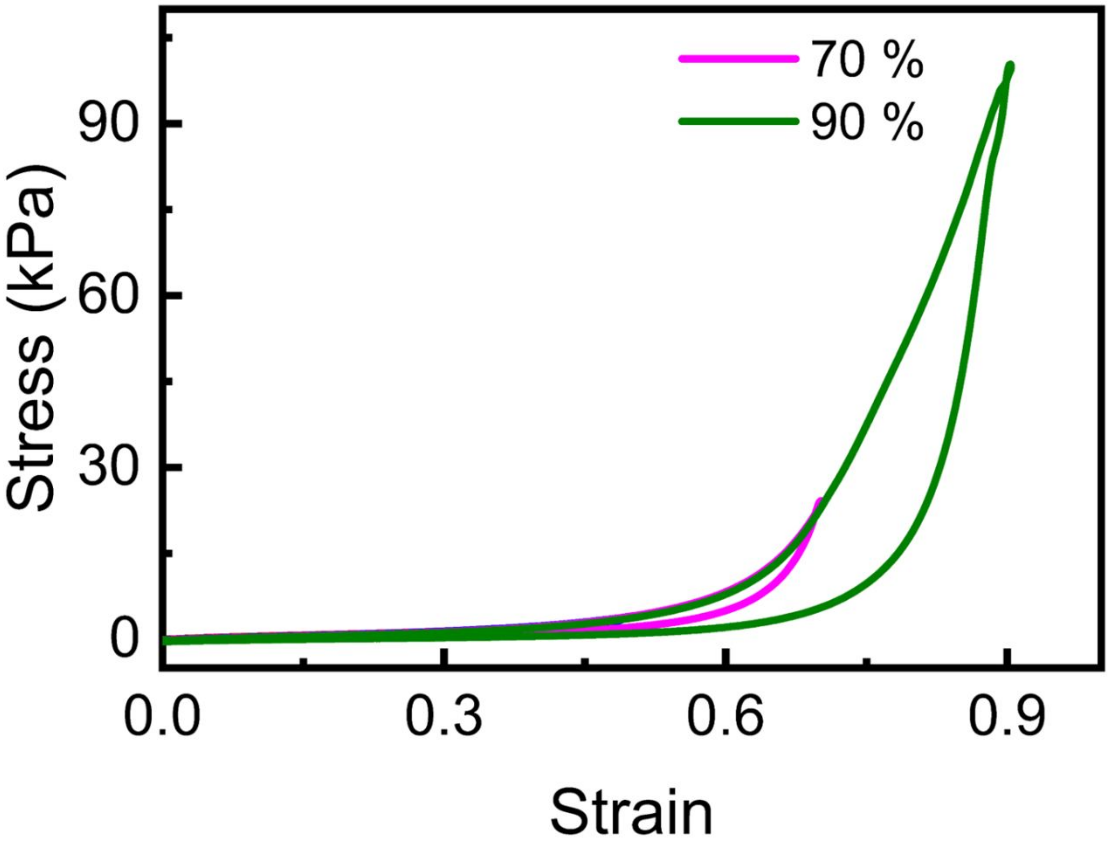

Figure S9. Stress−strain curves of CCS-rGO aerogel compressed at 70% and 90% compressive strain.

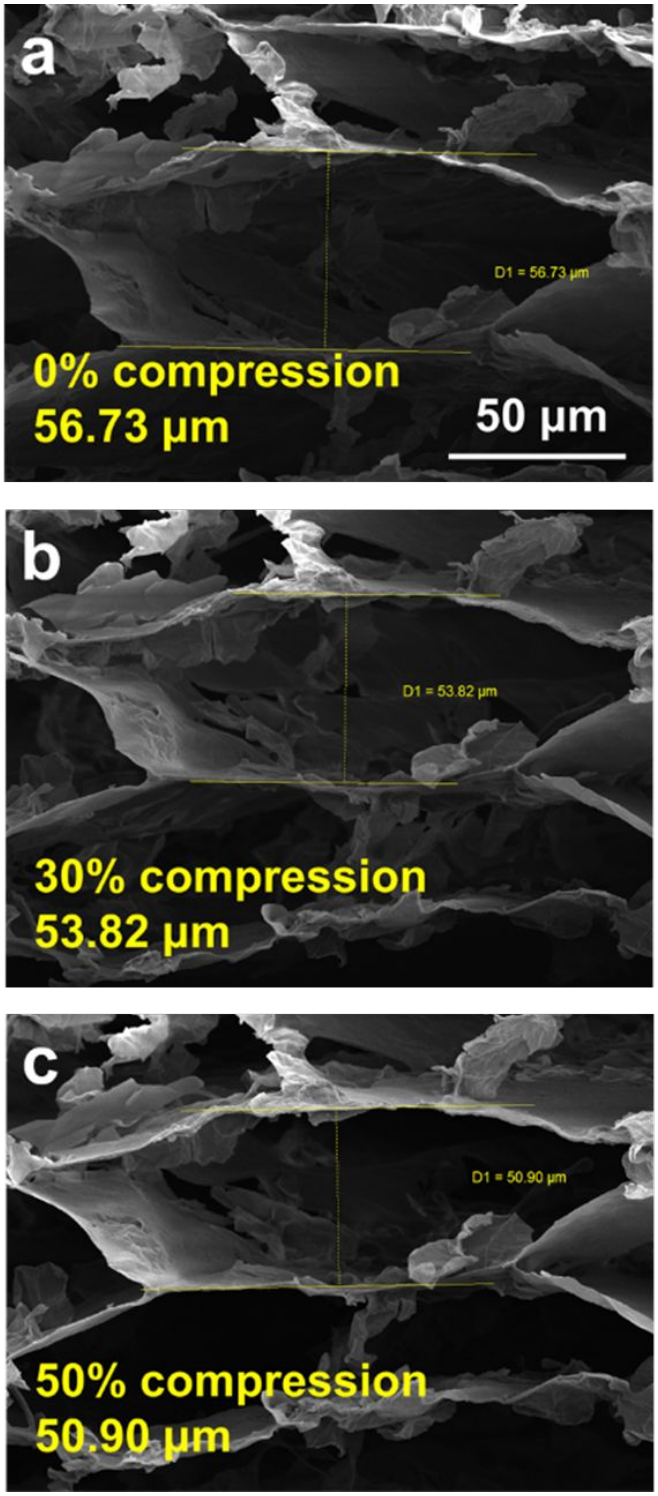

Figure S10. SEM images of CCS-rGO microstructure in a) 0%, b) 30%, and c) 50% compression strain.

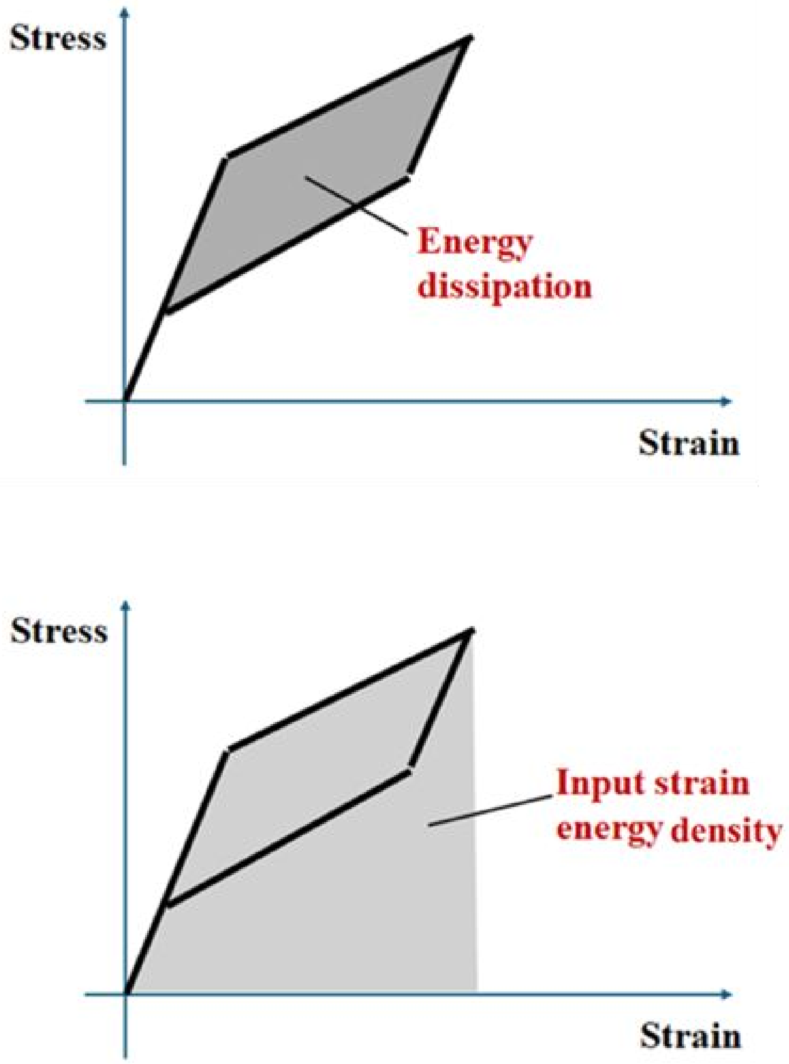

Figure S11. Illustration of the energy dissipation in compression cycles.

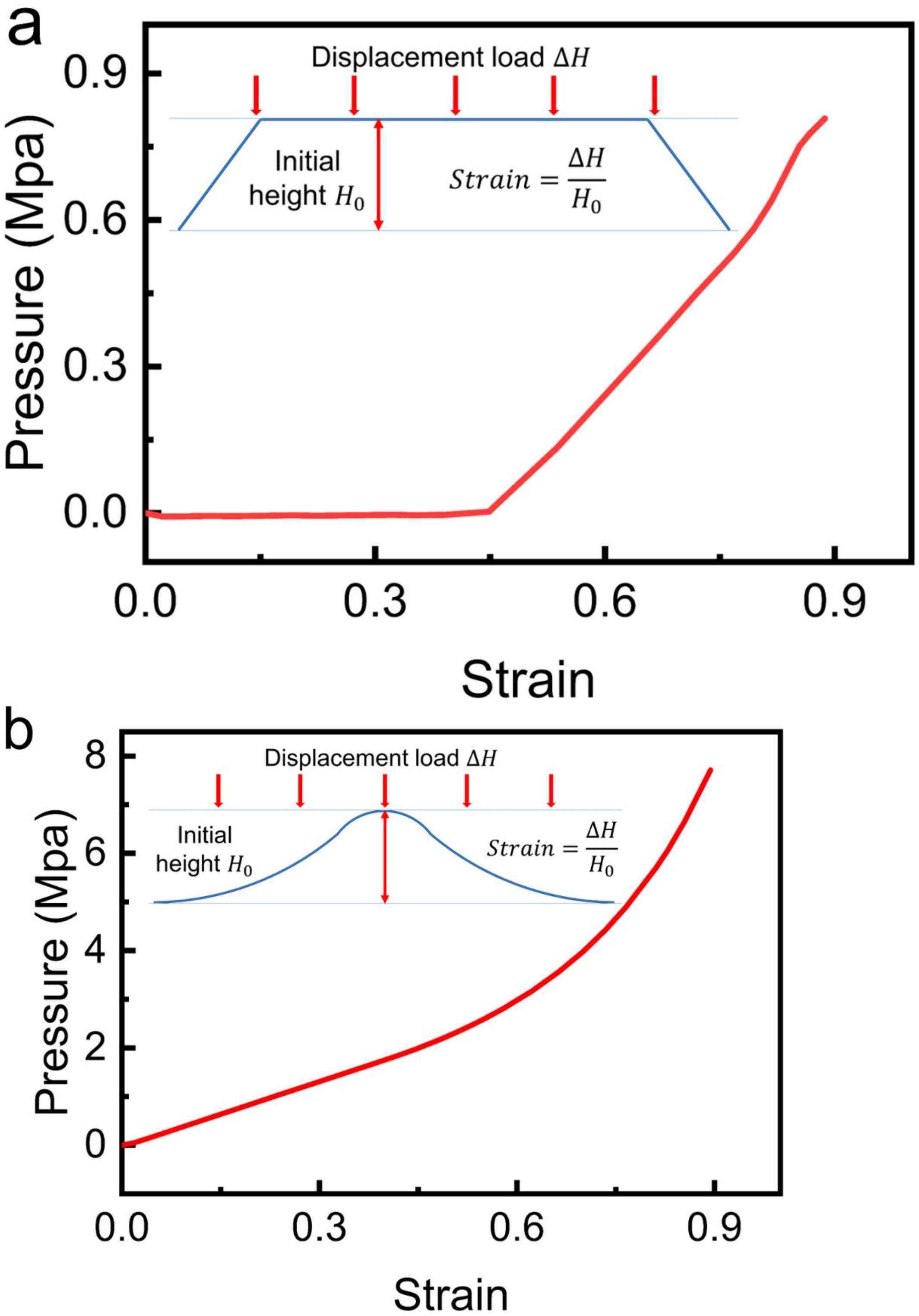

Figure S12. Configuration and results of 2D simulations for strain-pressure analysis. a) The original honeycomb and b) the reconfigured arch microstructure.

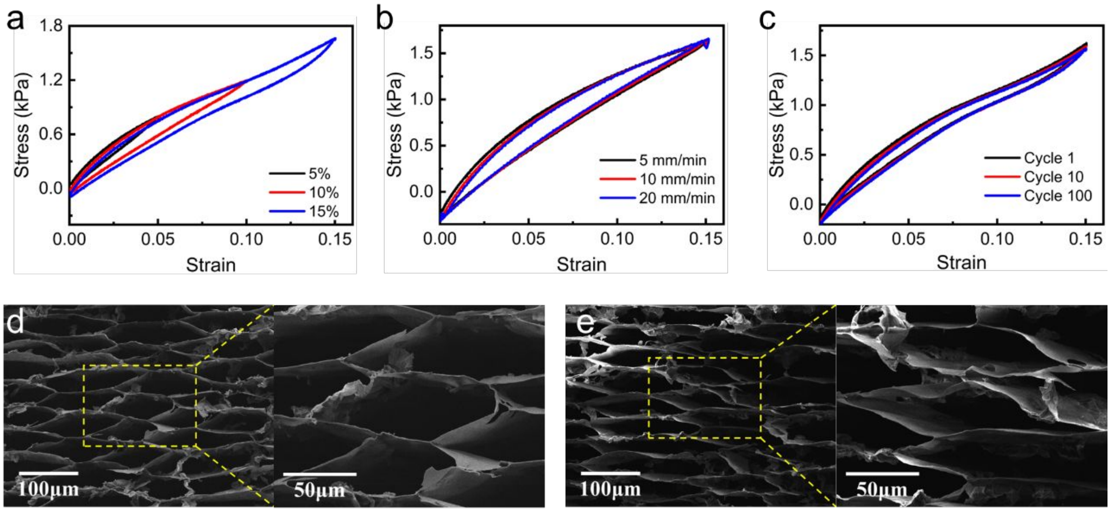

Figure S13. Tensile properties of CCS-rGO aerogel at radial direction. a) Stress−strain plots of CCS-rGO at 5-15% tensile strain. b) Stress−strain plots of CCS-rGO at 15% tensile strain under different tensile speed. c) Stress−strain plots of CCS-rGO at 15% tensile strain for 100 cycles. SEM images of CCS-rGO aerogel d) before and e) after 15% strain for 100 cycles.

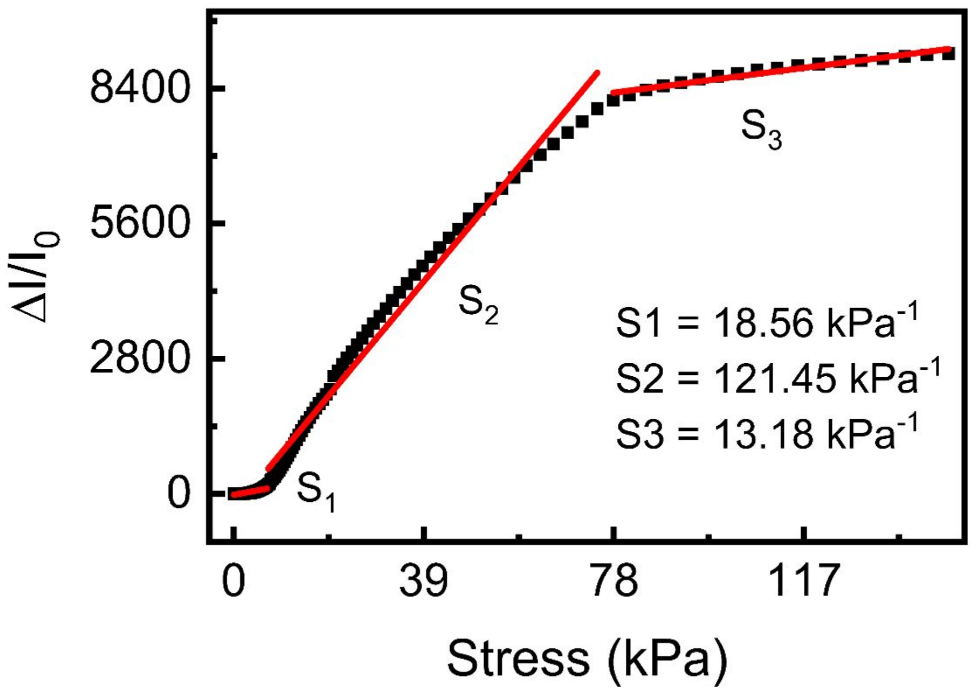

Figure S14. Sensitivity of CCS-rGO sensor at the full σ_{comp} range of 0-146.7kPa (corresponding ε_{comp} from 0 to -0.95).

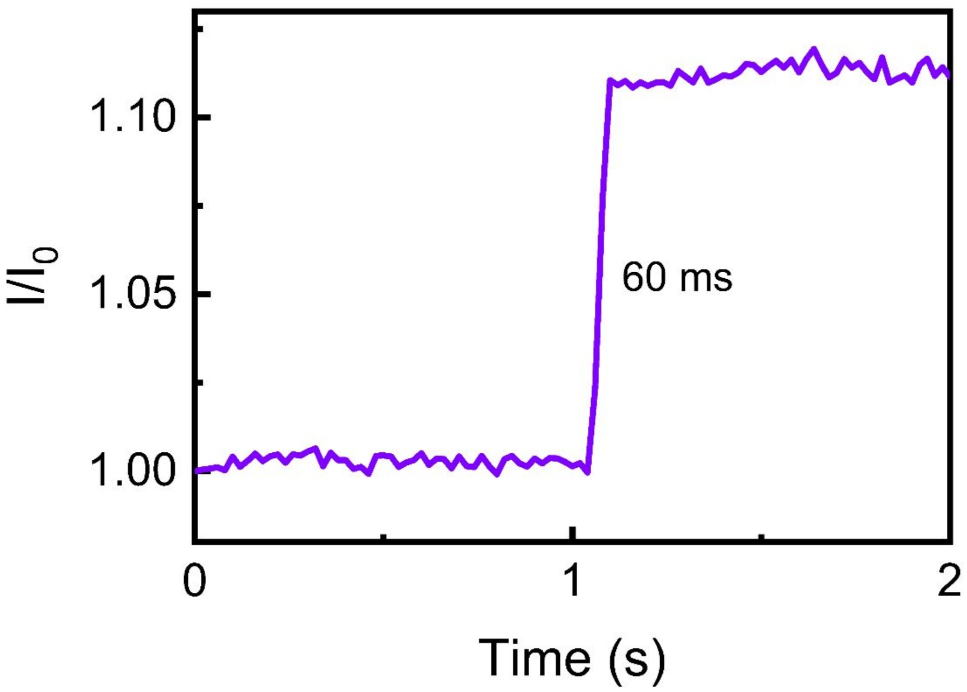

Figure S15. Response time of CCS-rGO sensor by the load of ~700 Pa and speed of 1m/s.

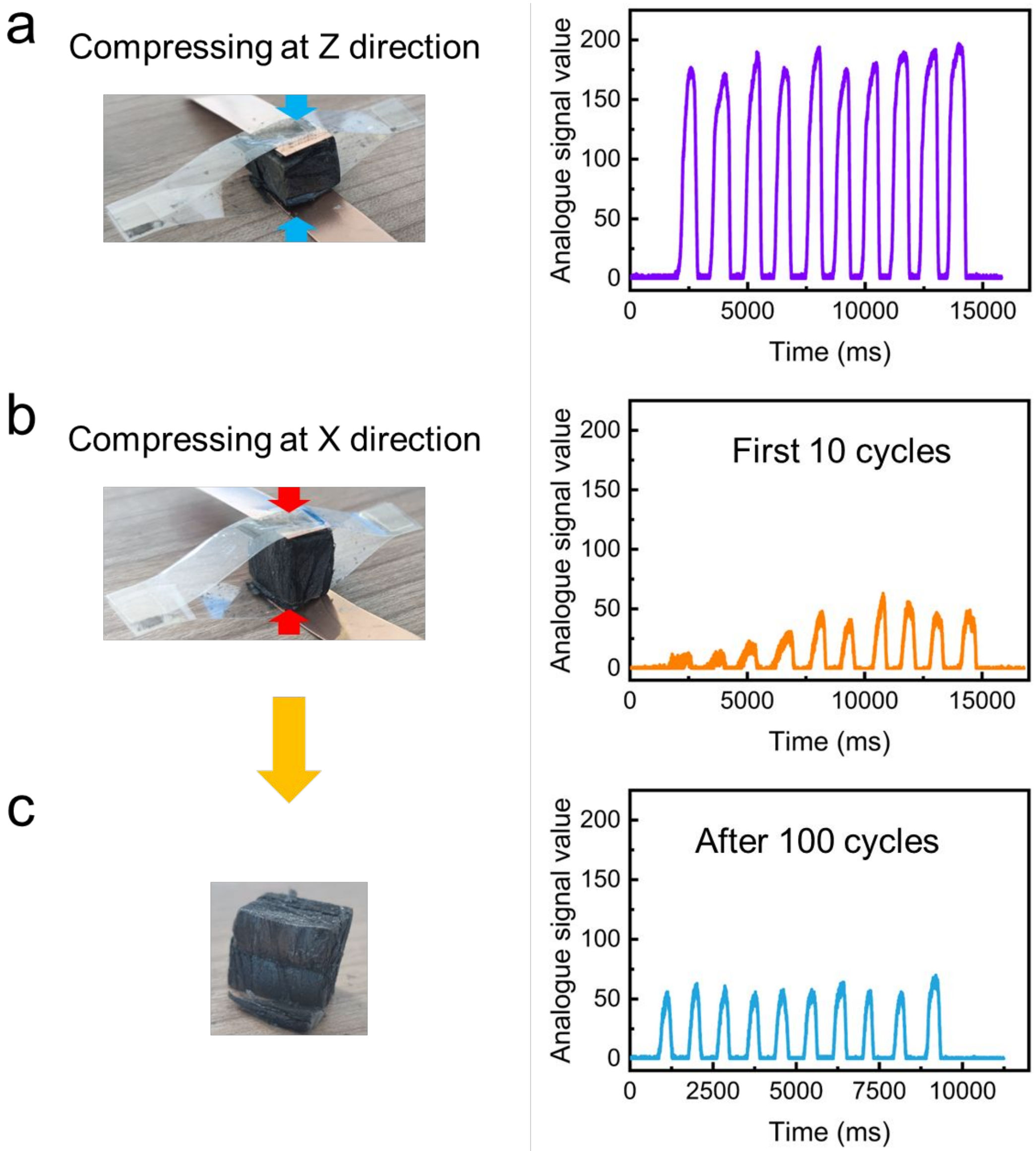

Figure S16. Failure test comparison of the CCS-rGO sensor at different compression directions. a) Configuration of compression in Z direction and corresponding analog signal curve. b) Configuration of compression in X direction and corresponding analog signal curve in first 10 cycles. c) Morphology of the sensor after 100 cycles in X direction and corresponding analog signal curve.

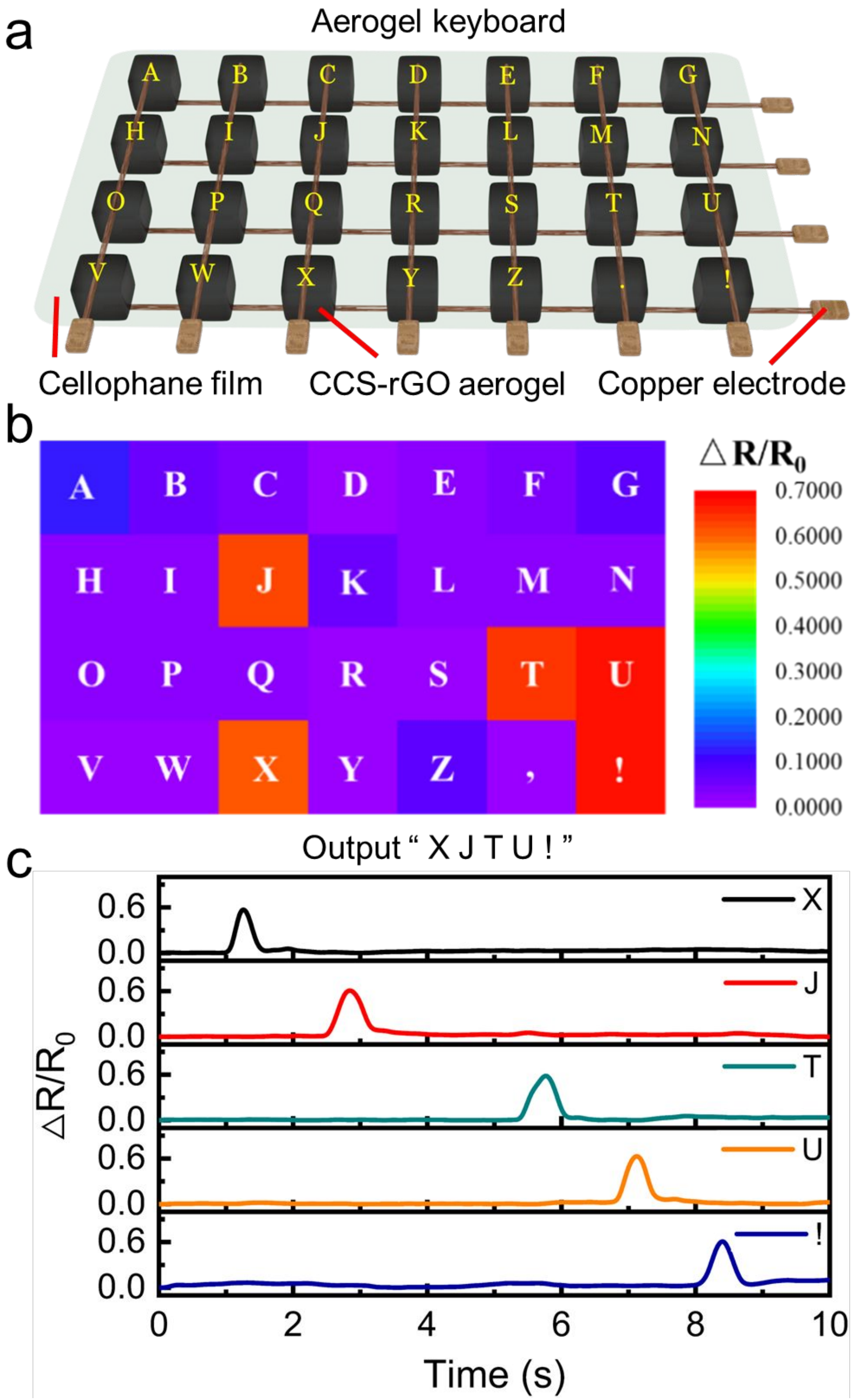

Figure S17. Tactical sensing keyboard by 28 CCS-rGO aerogels. a) Illustration of universal tactical sensing keyboard. b) Sensing heatmap of resistance changes for "XJTU!" output and c) corresponding resistance variation curve with time.

Supporting Table

	CS content	GA content
CCS-1 aerogel	1.2 wt. %	0.6 wt. %
CCS-2 aerogel	1.2 wt. %	1.2 wt. %
CCS-3 aerogel	1.2 wt. %	1.8 wt. %
CCS-4 aerogel	1.2 wt. %	2.4 wt. %
CCS-5 aerogel	0.8 wt. %	2.4 wt. %
CCS-6 aerogel	1.0 wt. %	2.4 wt. %
CCS-7 aerogel	1.2 wt. %	2.4 wt. %
CCS-8 aerogel	1.4 wt. %	2.4 wt. %

Table S1. CS and GA content of different CCS-aerogels.

Supporting Movies

Movie S1 - The deposition of water droplet on the surface of CS-GO aerogel
Movie S2 - FE Simulation on the thermal-mechanical deformation on a single beam
Movie S3 - A dynamic impact on CCS-rGO aerogel by a free-falling steel ball
Movie S4 - Demonstration of sensing accuracy and application as robotic hand control
Movie S5 - To play 'Twinkle, Twinkle, Little star' with CCS-rGO sensors based digital keyboard

REFERENCES

(1) Hummers, W. S., Jr.; Offeman, R. E. Preparation of Graphitic Oxide. *Journal of the American Chemical Society* **1958**, *80* (6), 1339-1339. DOI: 10.1021/ja01539a017.

(2) Torrisi, L.; Cutroneo, M.; Torrisi, A.; Silipigni, L. Measurements on Five Characterizing Properties of Graphene Oxide and Reduced Graphene Oxide Foils. *physica status solidi (a)* **2022**, *219* (6), 2100628. DOI: https://doi.org/10.1002/pssa.202100628.

(3) Teklu, A.; Barry, C.; Palumbo, M.; Weiwadel, C.; Kuthirummal, N.; Flagg, J. Mechanical Characterization of Reduced Graphene Oxide Using AFM. *Advances in Condensed Matter Physics* **2019**, *2019*, 8713965. DOI: 10.1155/2019/8713965.

(4) Wan, J.; Jiang, J.-W.; Park, H. S. Negative Poisson's ratio in graphene oxide. *Nanoscale* **2017**, *9* (11), 4007-4012, 10.1039/C6NR08657H. DOI: 10.1039/C6NR08657H.

(5) Wen, Y.; Gao, E.; Hu, Z.; Xu, T.; Lu, H.; Xu, Z.; Li, C. Chemically modified graphene films with tunable negative Poisson's ratios. *Nature Communications* **2019**, *10* (1), 2446. DOI: 10.1038/s41467-019-10361-3.

(6) Naik, G.; Krishnaswamy, S. Photoreduction and Thermal Properties of Graphene-Based Flexible Films. *Graphene* **2017**, *06* (02), 27-40. DOI: 10.4236/graphene.2017.62003.

(7) Chen, J.; Li, L. Thermal Conductivity of Graphene Oxide: A Molecular Dynamics Study. *JETP Letters* **2020**, *112* (2), 117-121. DOI: 10.1134/S0021364020140015.

(8) Su, Y.; Wei, H.; Gao, R.; Yang, Z.; Zhang, J.; Zhong, Z.; Zhang, Y. Exceptional negative thermal expansion and viscoelastic properties of graphene oxide paper. *Carbon* **2012**, *50* (8), 2804-2809. DOI: https://doi.org/10.1016/j.carbon.2012.02.045.

(9) Mahanta, N. K.; Abramson, A. R. Thermal conductivity of graphene and graphene oxide nanoplatelets. In *13th InterSociety Conference on Thermal and Thermomechanical Phenomena in Electronic Systems*, 30 May-1 June 2012, 2012; pp 1-6. DOI: 10.1109/ITHERM.2012.6231405.

(10) Sumarokov, V. V.; Jeżowski, A.; Szewczyk, D.; Dolbin, A. V.; Vinnikov, N. A.; Bagatskii, M. I. The low-temperature specific heat of thermal reduced graphene oxide. *Low Temperature Physics* **2020**, *46* (3), 301-305. DOI: 10.1063/10.0000703 (acccessed 2/26/2024).
